# Inteligencia artificial y salud digital en Colombia: panorama de los avances más recientes y retos futuros

**DOI:** 10.7705/biomedica.8248

**Published:** 2025-12-10

**Authors:** Diego M. López, Juan Sebastián Osorio

**Affiliations:** 1 Fellow, International Academy of Health Sciences Informatics - International Medical Informatics Association, IAHSI-IMIA, Genève, Switzerland Profesor, Facultad de Ingeniería Electrónica y Telecomunicaciones, Facultad de Ciencias de la Salud, Universidad del Cauca, Popayán, Colombia Universidad del Cauca Facultad de Ingeniería Electrónica y Telecomunicaciones, Facultad de Ciencias de la Salud Universidad del Cauca Popayán Colombia; 2 Consultant, I-TECH, Department of Global Health, University of Washington, Seattle, WA, USA Project manager, MIT Critical Data, Cambridge, MA, USA Dirección de Imágenes Médicas e IA en Salud, Centro de Biociencias, Ayudas Diagnósticas SURA University of Washington University of Washington Centro de Biociencias, Ayudas Diagnósticas SURA Seattle WA USA

La inteligencia artificial -impulsada por los recientes avances en inteligencia artificial generativa- ha demostrado tener una capacidad sin precedentes para facilitar las tareas cotidianas, desde la interacción con las personas simulando un interlocutor humano, hasta la creación de contenidos de multimedia y la automatización de procesos que parecían imposibles hasta hace unos pocos años. En el campo de la salud, la inteligencia artificial ha demostrado su potencial para impactar la investigación biomédica y la práctica clínica: tecnologías como el aprendizaje automático, el aprendizaje profundo y, más recientemente, los modelos generativos de lenguaje y los agentes inteligentes, están fortaleciendo paulatinamente los procesos de diagnóstico, pronóstico, personalización de tratamientos y gestión de datos clínicos ^1^^-^^2^.

En Colombia, como en otras naciones de ingresos bajos y medios, la inteligencia artificial se perfila como una de las tecnologías más prometedoras para mejorar la eficiencia y la calidad de la atención en salud. Diferentes actores, como los prestadores de servicios, los aseguradores, las entidades territoriales, las empresas de tecnología, los centros de investigación y las universidades, han desarrollado iniciativas y productos innovadores basados en la salud digital. Sin embargo, la transición de las experiencias piloto a la implementación clínica efectiva continúa enfrentando múltiples barreras debido a la complejidad y a las particularidades de nuestros sistemas de salud. López *et al*. ^3^ identificaron varios retos del uso de la inteligencia artificial en salud en los países de ingresos bajos y medios, los cuales agruparon en ocho categorías principales:

1. *Calidad de los datos*: precisión, coherencia, credibilidad, disponibilidad y diversidad de las fuentes utilizadas para entrenar modelos confiables de inteligencia artificial que sean representativos del contexto local.
2. *Conciencia del contexto*: capacidad para adaptar y validar los modelos de inteligencia artificial con datos locales, garantizando su pertinencia epidemiológica, su generalización adecuada y su capacidad de explicar los resultados.
3. *Marcos reguladores y legales*: acuerdos que definan las condiciones para proteger la privacidad, la seguridad y la equidad en el uso de la información, así como la responsabilidad y la gobernanza ética para una implementación segura y justa.
4. *Educación y resistencia al cambio*: formación y sensibilización de todos los actores del ecosistema de salud para facilitar la adopción de la inteligencia artificial.
5. *Metodología, investigación y desarrollo:* estandarización del diseño, reporte y evaluación de las intervenciones de la inteligencia artificial, incorporando enfoques centrados en el usuario y mecanismos de certificación de calidad.
6. *Recursos financieros:* priorización adecuada, financiamiento sostenido y estrategias de inversión para el desarrollo y el mantenimiento de las tecnologías de inteligenia artificial.
7. *Infraestructura y conectividad:* mejorar la conectividad, la capacidad computacional y la integración de los sistemas electrónicos de salud, y garantizar la interoperabilidad y el acceso seguro a los datos.
8. *Escalabilidad:* desarrollo de soluciones escalables, evaluación continua de su impacto y costo-efectividad para una adopción amplia de la inteligencia artificial en salud.

Este suplemento temático de *Biomédica* presenta una colección de artículos que, no solo exponen ejemplos de aplicaciones innovadoras de la inteligencia artificial en diferentes escenarios de la salud, sino que, también, de manera explícita o implícita, abordan varios de los retos mencionados anteriormente, ofreciendo un panorama del estado actual de la investigación en nuestro país y orientando las investigaciones futuras en este campo.

## Panorama del estado actual de la investigación en inteligencia artificial en salud y desafíos futuros

Para este suplemento temático en medicina digital y la inteligencia artificial en salud, se recibieron 17 manuscritos, de los cuales, tras un riguroso proceso de evaluación por pares, 6 trabajos fueron seleccionados para su publicación. Los artículos aquí reunidos abordan, desde distintas perspectivas, las experiencias y los desarrollos relacionados con la implementación de la inteligencia artificial en el contexto colombiano. En conjunto, ofrecen una visión integral del avance de esta disciplina en el país. A continuación, se resumen los estudios y se analiza la contribución directa o indirecta de cada uno para enfrentar los retos previamente descritos.

1. *Advanced artificial intelligence in piRNA and PIWI-like protein research: A systematic review of recurrent neural networks, long short-term memory, and emerging computational techniques* por Reyes *et al.*

Este trabajo presenta una revisión sistemática de modelos de inteligencia artificial aplicados a la detección y análisis de moléculas piRNA y proteínas PIWI, componentes clave en la regulación génica y en los procesos de cáncer y enfermedades infecciosas. Los autores establecen que los principales modelos reportados en la literatura corresponden a redes neuronales recurrentes, redes con memoria a corto plazo prolongada *(long short-term memory,* LSTM) y redes convolucionales de grafos (*global cycling network,* GCN).

La revisión expone desafíos críticos como la falta de transparencia y la reproducibilidad de los modelos, además de la gran demanda de infraestructura computacional, aspectos que limitan el avance de la bioinformática en los países de ingresos bajos y medios, y ponen de relieve la necesidad de fortalecer las capacidades locales en investigación computacional avanzada.

2. *Aplicación de la inteligencia artificial en el diagnóstico de alteraciones de la citología cervicouterina: estudio observacional en población universitaria por* Manzano-Chaya *et al.*

Este artículo presenta un estudio observacional en el nororiente colombiano para evaluar la capacidad discriminativa de cuatro modelos de aprendizaje profundo (DenseNet, InceptionV3, MobileNet y VGG19) en la detección de anomalías en las citologías cervicouterinas. Los autores aplican técnicas de transferencia de aprendizaje para optimizar el entrenamiento de los modelos locales y utilizan Grad-CAM como herramienta de interpretación visual, contribuyendo así a la transparencia y a la capacidad de explicación del sistema. Los resultados, que evidencian gran sensibilidad y especificidad, sugieren un enfoque costo-efectivo y escalable con potencial para fortalecer los programas de tamizaje y prevención en salud pública en el contexto colombiano.

3*. Maternal care: Is it good to trust clinical guidelines-based recommendations given by artificial intelligence?* por Pérez *et al.*

Los autores evalúan diferentes modelos generativos de lenguaje a gran escala (*large language model*, LLM), utilizados como herramientas de apoyo clínico en ginecología y obstetricia. Para garantizar que las recomendaciones generadas por estos modelos sean relevantes y estén adaptadas al contexto local, los autores implementaron un enfoque de generación aumentada por recuperación (*retrieval-augmented generation*, RAG) que integra la información proveniente de las guías clínicas colombianas.

El estudio destaca la importancia de la conciencia de contexto y de la formación de profesionales de la salud en el uso de estas tecnologías, evidenciando el potencial de la inteligencia artificial generativa para enriquecer la práctica médica y la necesidad de mantener la responsabilidad humana en la toma final de las decisiones clínicas.

4. *Sistema de apoyo a las decisiones clínicas basado en las reglas para la clasificación automatizada de la anemia en pacientes en hemodiálisis* por Tenorio *et al.*

En este trabajo se describe el desarrollo de un sistema de apoyo a la decisión clínica (*Clinical Decision Support System,* CDSS) orientado a la clasificación automatizada de la anemia de los pacientes en hemodiálisis. La solución extrae y estructura los datos provenientes del sistema de información de laboratorio (*library information systems*, LIS), utilizando los estándares HL7 y las guías regionales de la Sociedad Latinoamericana de Nefrología e Hipertensión, adaptadas al contexto de Colombia. Este sistema demuestra su utilidad en la práctica nefrológica al facilitar la estandarización de los criterios diagnósticos y fortalecer la calidad, la confiabilidad y la interoperabilidad de la información clínica, con lo cual contribuye a una atención más eficiente.

5. *Datos sintéticos en el contexto colombiano de un modelo de datos común para las aplicaciones de inteligencia artificial en salud materna: reporte de experiencia* por Torres-Silva *et al.*

Los autores presentan una experiencia pionera en la generación de datos sintéticos ajustados a la demografía y las guías clínicas de Colombia, con el objetivo de mitigar el problema de la escasez de datos locales disponibles para investigaciones en inteligencia artificial. Los autores emplean el modelo de datos común (*Observational Medical Outcomes Partnership Common Data Model*, OMOP-CDM) para estructurar y estandarizar la información. El artículo aborda directamente los desafíos de calidad, acceso y privacidad de los datos clínicos, y resalta el potencial de los datos sintéticos como estrategia viable para impulsar el desarrollo de los modelos de inteligencia artificial en salud materna, dentro de un marco ético y seguro.

6. *Clasificación de la expresión del receptor 2 del factor de crecimiento epidérmico humano (HER2) en tejido mamario cancerígeno por medio de inteligencia artificial* por Villota *et al*.

Este trabajo presenta el desarrollo de un modelo de inteligencia artificial para la clasificación automática de la expresión del receptor HER2, un biomarcador fundamental en el diagnóstico y tratamiento del cáncer de mama. Los autores construyeron una base de datos local con más de 28.000 imágenes histológicas, y usaron modelos de redes neuronales profundas y modelos transformadores (*transformers*) de visión, junto con transferencia de aprendizaje y técnicas de ensamble. El estudio destaca su validación con patólogos colombianos, su adecuación a marcos éticos y normativos nacionales, y el desarrollo de una plataforma intuitiva que facilita la interacción y la experiencia útil del usuario clínico, evidenciando el potencial de la inteligencia artificial para apoyar la oncología de precisión en los entornos locales.

## Conclusiones

Los trabajos aquí presentados reflejan la diversidad de capacidades y el creciente potencial del uso de la inteligencia artificial en salud en el contexto colombiano. Aunque existen múltiples experiencias adicionales no reportadas en este suplemento -muchas aún en las fases iniciales de desarrollo-, el conjunto de manuscritos reunidos evidencia un ecosistema en consolidación, con avances significativos en la investigación aplicada y en la adaptación de tecnologías al entorno local.

Para que estas soluciones puedan integrarse de manera segura en la práctica clínica, debe primero demostrarse -mediante evidencia científica sólida- su eficacia y seguridad en el uso con seres humanos. Por lo tanto, se necesita que toda investigación aplicada a la salud se rija con el mismo rigor metodológico y ético que caracteriza a la investigación biomédica.

En este sentido, resulta fundamental que el diseño y el reporte de las investigaciones en este campo, se ciñan a los estándares internacionales reconocidos. Guías como CONSORT-AI ^4^ y SPIRIT-AI ^5^ para los estudios clínicos, TRIPOD-AI ^6^ para los modelos de predicción, y DECIDE-AI ^7^ para los sistemas de soporte a las decisiones, constituyen herramientas esenciales para garantizar la transparencia, reproducibilidad y validez de los estudios.

En varios de los artículos que hacen parte de este suplemento, se hace referencia a estas guías, las cuales fueron, además, consideradas como marco de evaluación y retroalimentación para los autores durante el proceso editorial, con lo cual se reafirma el compromiso de *Biomédica* con la calidad y el fortalecimiento de la investigación en inteligencia artificial en salud.
